# Germany’s fourth COVID-19 wave was mainly driven by the unvaccinated

**DOI:** 10.1038/s43856-022-00176-7

**Published:** 2022-09-16

**Authors:** Benjamin F. Maier, Marc Wiedermann, Angelique Burdinski, Pascal P. Klamser, Mirjam A. Jenny, Cornelia Betsch, Dirk Brockmann

**Affiliations:** 1 Institute for Theoretical Biology and Integrated Research Institute for the Life-Sciences, Humboldt-University of Berlin, Philippstr. 13, 10115 Berlin, Germany; 2 Robert Koch Institute, Nordufer 20, 13353 Berlin, Germany; 3 University of Erfurt, Nordhäuserstr. 63, 99089 Erfurt, Germany; 4 Harding Center for Risk Literacy, University of Potsdam, Virchowstrasse 2-4, 14482 Potsdam, Germany; 5 Max Planck Institute for Human Development, Lentzeallee 94, 14195 Berlin, Germany; 6 Bernhard-Nocht-Institut, Bernhard-Nocht-Straße 74, 20359 Hamburg, Germany

**Keywords:** Viral infection, Computational biology and bioinformatics

## Abstract

**Background:**

While the majority of the German population was fully vaccinated at the time (about 65%), COVID-19 incidence started growing exponentially in October 2021 with about 41% of recorded new symptomatic cases aged twelve or above being symptomatic breakthrough infections, presumably also contributing to the dynamics. So far, it remained elusive how significant this contribution was and whether targeted non-pharmaceutical interventions (NPIs) may have stopped the amplification of the crisis.

**Methods:**

We develop and introduce a contribution matrix approach based on the next-generation matrix of a population-structured compartmental infectious disease model to derive contributions of respective inter- and intragroup infection pathways of unvaccinated and vaccinated subpopulations to the effective reproduction number and new infections, considering empirical data of vaccine efficacies against infection and transmission.

**Results:**

Here we show that about 61%–76% of all new infections were caused by unvaccinated individuals and only 24%–39% were caused by the vaccinated. Furthermore, 32%–51% of new infections were likely caused by unvaccinated infecting other unvaccinated. Decreasing the transmissibility of the unvaccinated by, e. g. targeted NPIs, causes a steeper decrease in the effective reproduction number ${{{{{{{\mathcal{R}}}}}}}}$ than decreasing the transmissibility of vaccinated individuals, potentially leading to temporary epidemic control. Reducing contacts between vaccinated and unvaccinated individuals serves to decrease ${{{{{{{\mathcal{R}}}}}}}}$ in a similar manner as increasing vaccine uptake.

**Conclusions:**

A minority of the German population—the unvaccinated—is assumed to have caused the majority of new infections in the fall of 2021 in Germany. Our results highlight the importance of combined measures, such as vaccination campaigns and targeted contact reductions to achieve temporary epidemic control.

## Plain language summary

With about 65% of its citizens vaccinated at the time, Germany experienced a large wave of COVID-19 in the fall of 2021, regionally overburdening the healthcare system. We are interested in how much this crisis was driven by infections in vaccinated versus unvaccinated people. We use a mathematical model to show that transmission of the disease during this period was largely driven by the unvaccinated population, despite representing a smaller proportion of the overall population. Our results suggest that higher vaccine uptake, reduced mixing between vaccinated and unvaccinated people, and targeted contact-reduction measures would have been effective measures to control spread at the time. These findings may have implications for how we manage future waves of COVID-19 or other diseases.

## Abstract (web summary)

Maier et al. develop a mathematical model to examine the contributions of vaccinated vs. unvaccinated populations to the wave of SARS-CoV-2 infections in Germany in autumn 2021. They report that the unvaccinated population were the main drivers of transmission and that targeted non-pharmaceutical interventions would likely have mitigated this.

## Introduction

Vaccines are the most powerful pharmaceutical tool to prevent infections with SARS-CoV-2 and combat the COVID-19 pandemic. Fast vaccine uptake by as many individuals as possible saves lives, people’s health, and livelihoods. Despite large-scale vaccine roll-out campaigns, many countries, most prominently in Europe, have experienced a rise in case numbers in the late summer and early fall of 2021 and reported effective reproduction numbers ${{{{{{{\mathcal{R}}}}}}}}$ above one for an extended period of time^1^. This means that on average, every infected person infected more than one other person, thus causing exponentially rising incidences^2^. Since the beginning of this pandemic, such resurgences have, in part, been mitigated by harsh non-pharmaceutical interventions (NPIs) such as lockdowns or curfews that limit the population’s contacts, thereby decreasing the effective reproduction number and relieving overburdened public health systems^3,4^. Measures that affect large parts of the general population over a long period of time can have devastating effects, such as increasing social inequality and domestic violence, detrimental impacts on mental health, or economic disruptions^5–9^. Such harsh restrictions should therefore be considered a last resort of pandemic control.

During the onset of the fourth COVID-19 wave in Germany, many hospitals and intensive care units (ICUs) were operating at maximum capacity or were projected to do so at a later point^10^. In the four weeks between Oct 11, 2021, and Nov 7, 2021, Germany’s central public health institute, the Robert Koch Institute (RKI) reported 250,552 new symptomatic infections in individuals with known vaccination status, 90,471 of which were attributed to vaccinated individuals, i.e. 36% were symptomatic breakthrough cases (41% in age groups eligible for vaccination)^11,12^. During this time, the average vaccination rate in different age groups [0,12), [12,18), [18,60), and 60+ were 0%, 40.1%, 72.4%, and 85.1%, respectively, leading to 0%, 4.8%, 41.6%, and 61.9% of new cases being classified as symptomatic breakthrough cases within the respective age groups^11^, Table 1. Simultaneously, the effective reproduction number remained at a relatively stable value of ${{{{{{{\mathcal{R}}}}}}}}\approx 1.2$ (under the assumption of a generation time of four days)^13^.

**Table 1:** Share of breakthrough infections in the age groups eligible for vaccination according to official estimates by the Robert Koch Institute (RKI)^11^ and the model for “low efficacy”, “medium efficacy”, and “high efficacy” scenarios.

Age group	RKI report (symptomatic cases)	Model (“high eff.”)	Model (“medium eff.”)	Model (“low eff.”)
adolescents	4.8%	5.1%	21.1%	25%
adults	41.6%	42.3%	51.2%	57%
elderly	61.9%	61.5%	74.1%	77.4%

Given that breakthrough cases are a challenge both for communication and vaccine acceptance^14^ and that harsh NPIs may be illegitimate for vaccinated individuals, the above situation raises two important questions: How much does the unvaccinated population contribute to the infection dynamics despite being in the minority? And could targeted NPIs aiming at reducing the contacts of unvaccinated individuals temporarily and sufficiently suppress the infection dynamics such that harsh, large-scale NPIs could be avoided?

To address these questions, we establish the contribution matrix approach, a theoretical concept derived from the next-generation matrix framework^15^. The contribution matrix quantifies the contributions to ${{{{{{{\mathcal{R}}}}}}}}$ caused by the infection pathways from un-/vaccinated individuals to other un-/vaccinated individuals, considering the age and contact structure of the population, vaccination rates, as well as expected vaccine efficacies regarding susceptibility and transmission reductions, respectively. In its general form, it quantifies the contributions made by any combination of two subpopulations.

**Fig. 1:**
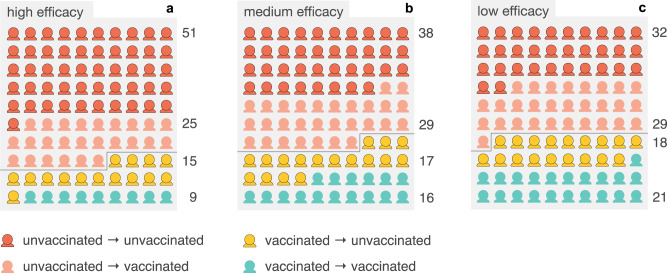
Estimated contributions of infection pathways towards new cases within vaccinated and unvaccinated subpopulations. Estimated contributions of infection pathways to ${{{{{{{\mathcal{R}}}}}}}}$ in the (**a**) “high efficacy”, (**b**) “medium efficacy”, and (**c**) “low efficacy” scenarios as a graphical representation of Tabs. 2–4. The charts can be read as follows: Consider an infected population that caused a new generation of 100 new infecteds. Then for (**a**), 51 of those newly infected individuals will be unvaccinated people that have been infected by other unvaccinated people. Likewise, 25 newly infected individuals will be vaccinated people that have been infected by unvaccinated individuals. Hence, 76 new infections will have been caused by the unvaccinated. Along the same line, 15 newly infecteds will be unvaccinated people that have been infected by vaccinated individuals and 9 newly infecteds will be vaccinated people that have been infected by other vaccinated individuals, totaling 24 new infections that have been caused by vaccinated individuals.

Based on this approach, we estimate that in October 2021, around 32%–51% (depending on vaccine efficacy) of the effective reproduction number was caused by unvaccinated individuals infecting other unvaccinated individuals (see Fig. 1). Since unvaccinated individuals have a higher probability of suffering from severe disease^16–18^, this contribution is the major factor that drove the public health system into a crisis characterized by hospitals and ICUs reaching maximum capacity. In contrast, we estimate that only 15%–18% of the reproduction number were attributable to vaccinated individuals infecting unvaccinated individuals. In October 2021, about 65% of the German population was fully vaccinated, implying that the majority of the overall population contributed little to the amplification of the crisis. In total, we estimate that the vaccinated population contributed 24%–39% to ${{{{{{{\mathcal{R}}}}}}}}$ while the unvaccinated population contributed the remaining 61%–76%, despite the fact that unvaccinated individuals have been in the minority in Germany. 9%–21% of new infections would be caused by vaccinated individuals infecting other vaccinated people. In total, we estimate that unvaccinated individuals were involved in 8–9 out of 10 new infections, either as infecting, acquiring infection, or both.

We further argue that regarding the situation in the fall of 2021, the unvaccinated would have had to reduce their transmissibility two to three times as strongly as the vaccinated in order for the system to reach ${{{{{{{\mathcal{R}}}}}}}}=1$ (and hence containment of the infection wave), if the burden of contact reductions were to be distributed between the two subpopulations according to their respective contributions. Moreover, decreasing mixing between individuals of distinct vaccination status can decrease ${{{{{{{\mathcal{R}}}}}}}}$. Ultimately, a higher vaccine uptake would have led to less unvaccinated being involved in infections, which can not only decrease ${{{{{{{\mathcal{R}}}}}}}}$, but is critical for relieving an overburdened public health system, as they are more likely to suffer from severe disease. Combinations of these interventions that address mainly the unvaccinated might have rendered the dynamics subcritical.

## Methods

### Mathematical framework

We use a population-structured compartmental infectious disease model that captures a variety of aspects regarding vaccination against COVID-19 (see Supplementary Methods, Sec. 1.1.1). The model’s dynamics are fully described by the next-generation matrix *K*_*ji*_ of small domain (see Supplementary Methods, Sec. 1.1.2), which quantifies the average number of offspring in group *j* caused by a single infectious individual in group *i*^15^. Here, the index *i* (or *j*, respectively) refers to the subpopulation that is determined by a respective age group and the vaccination status within that group, thus yielding two subpopulations per age group. In the regime of small outbreaks (relative to the total population size), the ordinary differential equations governing the epidemic growth can be linearized, with the dynamics being determined by *K*_*ji*_, such that the generational growth of the number of infected individuals in group *i* follows

1 $${y}_{j}(g+1)=\mathop{\sum}\limits_{i}{K}_{ji}{y}_{i}(g),\qquad g=0,1,2,...$$

The incidence approaches the eigenstate *y*_*i*_ of *K*_*ji*_ that corresponds its spectral radius, which in turn is equal to the effective reproduction number^15^. Hence, the entries of the normalized eigenvector ${\hat{y}}_{i}={y}_{i}\big/{\sum }_{j}{y}_{j}$ contain the relative frequency of newly infected individuals in age/vaccination group *i*.

Consequently, the number of *j*-offspring caused by *i*-individuals in a dynamical system defined by *K*_*ji*_ is given by the contribution matrix

2 $${C}_{ji}={K}_{ji}{\hat{y}}_{i}.$$

Summing over all matrix elements of *C*_*ji*_ yields the effective reproduction number ${{{{{{{\mathcal{R}}}}}}}}$ (see Supplementary Methods, Sec. 1.1.1)). A single matrix element *C*_*ji*_ can thus be considered the contribution of the *i* → *j* infection pathway to the reproduction number (a derivation of the concept and an operational definition of *C*_*ji*_ can be found in the Supplementary Methods, Sec. 1.1.1–1.1.2 and Sec. 1.2.4), respectively). The normalized contribution matrix *C*_*ji*_/${{{{{{{\mathcal{R}}}}}}}}$ gives the relative contributions of *i* → *j* infections towards ${{{{{{{\mathcal{R}}}}}}}}$ (and consequently, towards the total number of new infections).

We derive explicit equations for the contributions of un-/vaccinated individuals in the homogeneous case, i.e. ignoring age structure (see Supplementary Methods, Sec. 1.2.3). These contributions are

3 $${C}_{u\leftarrow u}=\frac{{(1-v)}^{2}}{1-vs}{{{{{{{{\mathcal{R}}}}}}}}}_{u}$$

4 $${C}_{u\leftarrow v}=\frac{v(1-v)(1-s)(1-r^{\prime} )}{1-vs}{{{{{{{{\mathcal{R}}}}}}}}}_{v}$$

5 $${C}_{v\leftarrow u}=\frac{v(1-v)(1-s)}{1-vs}{{{{{{{{\mathcal{R}}}}}}}}}_{u}$$

6 $${C}_{v\leftarrow v}=\frac{{v}^{2}{(1-s)}^{2}(1-r^{\prime} )}{1-vs}{{{{{{{{\mathcal{R}}}}}}}}}_{v},$$

where *v* is the vaccine uptake, *s* is the susceptibility reduction after vaccination, $r^{\prime} =1-(1-r)/b$ is the adjusted transmissibility reduction (i.e. it contains the relative increase of the recovery rate after a breakthrough infection *b* and viral shedding reduction *r*), ${{{{{{{{\mathcal{R}}}}}}}}}_{u}$ is the base transmissibility of unvaccinated infecteds, and ${{{{{{{{\mathcal{R}}}}}}}}}_{v}$ is the base transmissibility of vaccinated infecteds (both of which quantify differences in behavior in the respective groups). The total effective reproduction number is given by

7 $$\begin{array}{lll}{{{{{{{\mathcal{R}}}}}}}}&=&{C}_{u\leftarrow u}+{C}_{v\leftarrow u}+{C}_{u\leftarrow v}+{C}_{v\leftarrow v}\\ &=&(1-v){{{{{{{{\mathcal{R}}}}}}}}}_{u}+v(1-s)(1-r^{\prime} ){{{{{{{{\mathcal{R}}}}}}}}}_{v}.\end{array}$$

### Model structure, parameters, and scenarios

In the full model, we construct the next-generation matrix of small domain (see Supplementary Methods, Eq. (S3)) based on the following observations, assumptions, and estimates: We structure the population into four age groups [0,12) (children), [12,18) (adolescents), [18,60) (adults), and 60+ (elderly). Contact numbers between those age groups and subpopulation sizes were constructed based on the POLYMOD (2005) data set^19,20^ using the ‘socialmixr’ software package^21^ (see Supplementary Methods, Sec. 1.2.1). Since vaccine efficacy was, at the time of writing, estimated only for the status “fully vaccinated” in Germany without distinguishing between different vaccines, we solely distinguish between “unvaccinated” and “vaccinated” individuals in the model, regardless of the make of the received doses (note that by the fall of 2021, a total number of four vaccine types was available in Germany, i.e., Spikevax (Moderna), Ad26.COV2.S (Janssen), Vaxzevria (AstraZeneca), and Comirnaty (BioNTech/Pfizer) with the latter being by far the most used^22^). Following the example of Scholz et al.^23^, we further assume that children and adolescents have reduced susceptibility to the virus and a reduced base transmissibility if infected, as was observed in Germany, Israel, and Greece^24–26^. In the discussed time frame, 14.7%, 9.4%, 60.2%, and 15.7% of new cases can be attributed to the respective age groups [0,12), [12,18), [18,60), and 60+^12^. In order to match this distribution approximately, we calibrate the base susceptibility (i. e. susceptibility without vaccination) and infectiousness of our model by assuming that children are 72% as susceptible and 63% as infectious as adults (72% and 81% for adolescents), which is larger than what was observed for the wild type^25,26^, see Supplementary Methods, Sec. 1.2. However, since the B.1.617.2 variant (Delta) that was predominant in Germany in October/November 2021 was generally observed to be more infectious than the wild type^27^, such an increase is plausible. Note that in principle, heterogeneous ascertainment may lead to a distribution of detected cases that is skewed towards the adult population, as children and adolescents may have higher probability of suffering from an asymptomatic infection^28^ and thus are less likely to be detected via symptom-based testing strategies. Yet, by the fall of 2021, Germany made regular screening via rapid antigen tests mandatory in schools across the country, potentially lowering the level of under-ascertainment in these age groups^29^. Nevertheless, we test how our results change by assuming children and adolescents are as susceptible as adults in a sensitivity analysis (see Supplementary Methods, Sec. 1.3.3). Additionally, note that we ignore the number of recovered individuals. Until Oct 10, 2021, about 4.3 million infections were reported in Germany^13^, 74% of which likely received a vaccination^30–32^ and are therefore considered as vaccinated in our analysis. With an under-ascertainment ratio of about 1.8^33^, we estimate that the total number of non-vaccinated recovered individuals was on the order of 2.4% of the population in Germany at the time, and therefore negligible in our analysis.

In Germany, an estimated average vaccine efficacy of 72% against symptomatic COVID-19 in adults and the elderly was found for cases reported between Oct 11, 2021 and Nov 7, 2021^11^. Vaccine efficacy for adolescents was not reported due to the respective data being potentially unreliable (low number of cases). Because these efficacies were computed for symptomatic cases, we use their values as a “high efficacy” scenario regarding vaccine efficacy in our analysis, because unreported and/or asymptomatic breakthrough infections might lower the estimated efficacies (see Supplementary Methods, Sec. 1.2.5). However, note that these observed 72% vaccine efficacy are in line with an estimated population-wide vaccine efficacy against infection based on vaccination time series and waning immunity data that was published in a meta-review by the WHO^34,35^. In order to obtain breakthrough infection rates in adolescents on the order of observed symptomatic breakthrough cases we assume a vaccine efficacy of *s* = 92% for adolescents. Despite being comparably large, this value seems justified considering that adolescents have been made eligible to receive a vaccine in Germany only shortly prior to the study period, and a high vaccine efficacy against infection with the Delta variant has been reported for this age group^36^. Regarding the infectiousness of individuals suffering from breakthrough infections, viral load of vaccinated patients suffering from symptomatic COVID-19 was reported to be at the same level as of those unvaccinated^37,38^. Another study from the UK found decreased infectiousness in breakthrough infections^39^. Considering both these results, we assume a conservative transmission reduction of *r* = 10% for breakthrough infections. In agreement with the literature^37,40^ we further consider that the average infectious period of breakthrough infections is shorter than for unvaccinated individuals and assume a 50% increase in recovery rate for the vaccinated, amounting to an average infectious period that is 2/3 as long as that of unvaccinated infecteds (*b* = 3/2) (see Supplementary Methods, Sec. 1.2.2). Such an increased recovery rate can also be caused by deliberate behavior. As individuals that are not opposed to vaccination typically adhere to protection measures more consistently^41^, behavioral changes following a breakthrough infection might further decrease the effective infectious period. Note that together with a decreased duration of infection *b* = 3/2, the adjusted transmission reduction reads $r^{\prime} =1-(1-r)/b=40 \%$, which is lower than a 63% reduction that was observed for household transmissions of the Delta variant between infected vaccinated and susceptible unvaccinated individuals in the Netherlands in August and September 2021, close to our observational period^42^. As this reduction was observed to wane over time^43^, $r^{\prime} =40 \%$ is a reasonable assumption.

In a second, “medium efficacy” scenario, we consider that vaccine efficacies against infection are in the range of 50%–60%, i.e. lower than the observed value against symptomatic COVID-19, and lower than vaccine efficacies reported in the UK for the Comirnaty (BioNTech/Pfizer) vaccine^44^, considering that partial immunity might have waned over time^45^. Since vaccine efficacy is expected to decrease with age^45,46^, we assume an efficacy against infection of *s* = 60% for adolescents and adults as well as *s* = 50% for the elderly (see Supplementary Methods, Sec. 1.2.4).

Finally, we also discuss a “low efficacy” scenario where the susceptibility reduction is assumed to be much lower than the observed efficacy against symptomatic COVID-19, namely 50% for adolescents and adults, and 40% for the elderly (see Supplementary Methods, Sec. 1.2.5).

To summarize the main scenarios, for the “high efficacy” the vaccination efficacy *s* for adolescents, adults, and elderly is assumed to be 92%, 72%, 72%, in the “medium efficacy” scenario 60%, 60%, 50%, and in the “low efficacy” scenario 50%, 50%, 40%, respectively.

Based on these considerations we compute the respective full-model next generation matrices *K*_*ji*_ and numerically find the normalized population eigenvectors ${\hat{y}}_{i}$ corresponding to the respective and the contribution matrices *C*_*ji*_, which we further reduce to the two-dimensional vaccination status space by summing over the respective contributions of age groups (see Supplementary Methods, Eq. (S2)).

### Reporting summary

Further information on research design is available in the Nature Research Reporting Summary linked to this article.

## Results

As a first model validation we find that for the high efficacy scenario the relative size of breakthrough infections within age groups eligible for vaccination is in good agreement with the share of reported symptomatic breakthrough cases (Table 1), albeit being slightly larger than reported values, mirroring the fact that the official number of breakthrough infections is likely affected by underreporting^11^ and that the number of infections will be larger than the number of symptomatic breakthrough cases.

For all scenarios, we find that the largest entry in the contribution matrix is given by the unvaccinated → unvaccinated infection pathway, with a 51.4% (high efficacy), 38.1% (medium efficacy) and 31.6% (low efficacy) contribution respectively, see Tables 2, 3, 4 and Fig. 1. Most noteworthy, these numbers represent the largest contributions although the unvaccinated population is smaller than the vaccinated one. Moreover, the total contribution of the unvaccinated population to the effective reproduction number is 75.9%, 66.6%, and 61.1% for the high, medium, and low efficacy scenarios, respectively. In total, the unvaccinated population plays a role in 91.1% (high), 84% (medium), or 79.3% (low efficacy) of cases—either as infecting, acquiring infection, or both.

**Table 2:** Contribution to ${{{{{{{\mathcal{R}}}}}}}}$ from infections between vaccinated and unvaccinated populations for the upper parameter bounds.

	← (u)nvaccinated	← (v)accinated
u ←	51.4%	15.0%
v ←	24.5%	9.1%
total	75.9%	24.1%

**Table 3:** Relative contributions to ${{{{{{{\mathcal{R}}}}}}}}$ from infections between vaccinated and unvaccinated groups for the “medium efficacy” scenario.

	← (u)nvaccinated	← (v)accinated
u ←	38.1%	17.4%
v ←	28.5%	16.0%
total	66.6%	33.4%

**Table 4:** Relative contributions to ${{{{{{{\mathcal{R}}}}}}}}$ from infections between vaccinated and unvaccinated groups for the “low efficacy” scenario.

	← (u)nvaccinated	← (v)accinated
u ←	31.6%	18.2%
v ←	29.5%	20.7%
total	61.1%	38.9%

Since vaccine efficacy is expected to decrease with age and time passed after vaccination^45^, we test how our results for the “medium efficacy” scenario change when assuming a more pessimistic susceptibility reduction of 40% for the elderly while keeping 60% for all other age groups (see Supplementary Methods, Sec. 1.3.5). We find that our results do not change substantially (see Supplementary Table 3), which can be attributed to the fact that the elderly generally have a lower contact behavior than other age groups.

In order to test the validity of the homogeneous approach, we further use Eqs. (3)–(6)) to compute the contribution matrix with *v* = 65%, *s* = 72%, *r* = 10%, and *b* = 3/2, assuming ${{{{{{{{\mathcal{R}}}}}}}}}_{u}={{{{{{{{\mathcal{R}}}}}}}}}_{v}$. We find relative contributions of ${C}_{u\leftarrow u}/{{{{{{{\mathcal{R}}}}}}}}=50.1 \%$, ${C}_{v\leftarrow u}/{{{{{{{\mathcal{R}}}}}}}}=26.1 \%$, ${C}_{u\leftarrow v}/{{{{{{{\mathcal{R}}}}}}}}=15.7 \%$, ${C}_{v\leftarrow v}/{{{{{{{\mathcal{R}}}}}}}}=8.1 \%$, hence being in good agreement with the results of the age-structured model (cf. Tab. 2), showing that Eqs. (3)–(6)) can be used to estimate the order of magnitude of the contributions by the respective infection pathways. We expect this approximation to lose its validity for situations in which model assumptions become even more heterogeneous (e.g. strong differences in contact structure between age groups, vaccine uptakes per age group, or vaccine efficacy per age group).

**Fig. 2:**
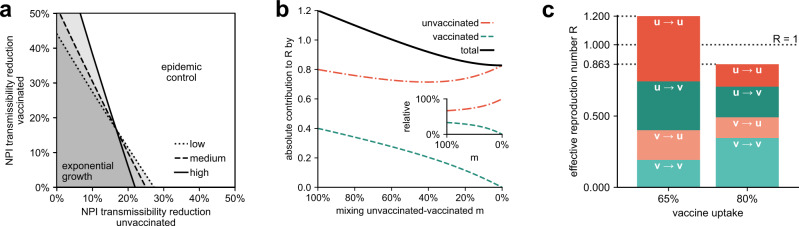
Efficacy of potential interventions to achieve temporary epidemic control. **a** Required additional transmissibility reduction for the unvaccinated (horizontal axis) and vaccinated (vertical axis) population to lower ${{{{{{{\mathcal{R}}}}}}}}$ to values below one, based on the assumption that the initial effective reproduction number is equal to ${{{{{{{\mathcal{R}}}}}}}}=1.2$. **b** The absolute contributions to ${{{{{{{\mathcal{R}}}}}}}}$ of the unvaccinated (orange) and vaccinated population (green) as well as their sum (black) with decreasing mixing *m* between both groups, based on the “medium efficacy” scenario. The inset shows the respective relative contributions. Note that if heterogeneous mixing was already present during our observational period, the monotonically increasing contribution of the unvaccinated displayed in the inset implies that our results of Fig. 1 are actually lower bounds of the true contribution. **c** Absolute contributions to ${{{{{{{\mathcal{R}}}}}}}}$ for infections between and across groups of vaccinated and unvaccinated individuals at the vaccine uptake during the observational period (left bar) and a hypothetical vaccine uptake of 80% in the total population, i.e., 90% in the age groups that were, at the time, eligible for vaccination (right bar), based on the “medium efficacy” scenario. The latter would have sufficed to suppress ${{{{{{{\mathcal{R}}}}}}}}$ sufficiently below one, assuming that other factors determining the base transmissibility remained on the same level.

During the period of time when vaccine efficacies were measured^11^, the reproduction number in Germany was reported to be at a relatively stable value of ${{{{{{{\mathcal{R}}}}}}}}=1.2$^13^. In order to achieve temporary epidemic control, it is necessary to reach a value of ${{{{{{{\mathcal{R}}}}}}}} < 1$ for a substantial amount of time^2^. We therefore study how the effective reproduction number would change if the transmissibility of unvaccinated individuals would be reduced. This could, for instance, be achieved by strict enforcement of contact rules regarding unvaccinated individuals at private and public gatherings that were partially in place in Germany^47^. For our analysis we gauge *K*_*ji*_ such that ${\sum }_{ji}{C}_{ji}={{{{{{{\mathcal{R}}}}}}}}=1.2$ for either of the base scenarios and then individually scale the transmissibility of the vaccinated and unvaccinated to obtain those values at which the critical value ${{{{{{{\mathcal{R}}}}}}}}=1$ is attained, Fig. 2a. We find that a transmission reduction of 22%–27% in the unvaccinated population would suffice to reach ${{{{{{{\mathcal{R}}}}}}}}=1$ without the need for any further restrictions. In contrast, NPIs that would affect both, vaccinated and unvaccinated to the same degree, would need to cause more than 17% of transmissibility reduction across the entire population to achieve epidemic control. For completeness and to put numbers in perspective one may also consider the unlikely scenario where NPIs are only in place for the vaccinated population yielding a required transmissibility reduction of 43%–73% in that group to achieve epidemic control, highlighting that vaccinated individuals would have to decrease their transmissibility less strongly than unvaccinated individuals for a distribution of the burden of contact reductions that corresponds to their respective contributions. The way to reach ${{{{{{{\mathcal{R}}}}}}}}=1$ in the plane spanned by NPI-based transmissibility reductions in both respective subpopulations that acknowledges these contributions with appropriate weighting is given by the linear function that is perpendicular to the isoclines shown in Fig. 2a. Using the fact that the homogeneous model given by Eqs. (3)–(6) yields acceptable approximations to the full model, we use Eq. (7) to derive the slope $\chi =v(1-s)(1-r^{\prime} )/(1-v)$ of this function (see Supplementary Methods, Sec. 1.3.2). This quantity has to be read as “if the unvaccinated population reduces its transmissibility by 10%, the vaccinated population has to reduce its transmissibility by *χ* × 10% in order for the system to quickly approach ${{{{{{{\mathcal{R}}}}}}}}=1$”. With *v* = 65%, *s* = 72%, $r^{\prime} =40 \%$ for the “high efficacy” scenario, as well as *s* = 60% for the “medium” and *s* = 50% for the “low efficacy” scenario, we find *χ* = 0.31, *χ* = 0.45, and *χ* = 0.55, respectively, which suggests that in order to adequately distribute the burden of further transmissibility reductions between the respective subpopulations, unvaccinated individuals would have to reduce their transmissibility two to three times as strongly as the vaccinated population.

**Fig. 3:**
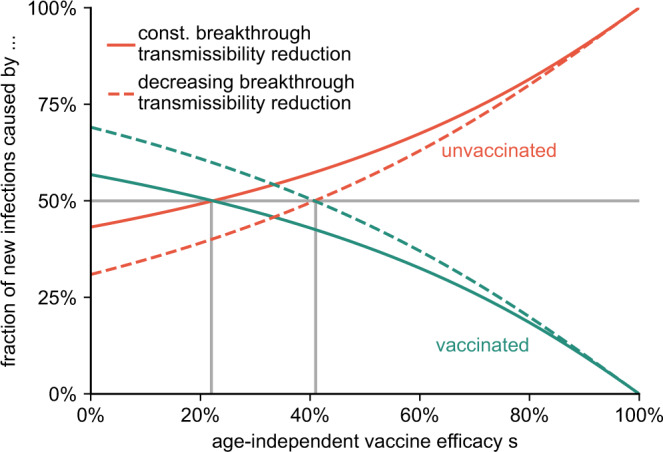
Fraction of new cases caused by the unvaccinated and vaccinated population for varying age-independent vaccine efficacy *s*. We consider an optimistic scenario with constant *r* = 0.1 and *b* = 3/2 (solid lines), and a pessimistic estimation in which *r* and *b* decrease according to *r* = *s*/10 and *b* = *s*/2 + 1 (dashed lines). As long as *s* remains larger than approximately 22% (optimistic, $r^{\prime} =40 \% ^{\prime}$) or 41% (pessimistic, $r^{\prime} =20 \%$), the unvaccinated minority still causes the majority of infections, see also Supplementary Methods, Sec. 1.3.1 and Sec. 1.3.7.

We further test the robustness of our results regarding vaccine efficacy by varying an age-independent vaccine efficacy against infection that ranges from *s* = 100% to *s* = 0%, (i) leaving *r* = 10% and *b* = 3/2 unchanged as an optimistic estimation and (ii) proportionally scaling *r* = *s*/10 and *b* = *s*/2 + 1 as a pessimistic estimation, while assuming vaccine uptake as reported in the Methods section (see Supplementary Methods, Sec. 1.3.1). We find a monotonic decrease of breakthrough infections from non-zero values for *s* = 0% to zero for *s* = 100%. Notably, we find that as long as vaccine efficacies do not drop below 22% (optimistic) or 41% (pessimistic), the majority of new cases remains to be caused by the minority of the population, which are the unvaccinated (see Fig. 3 and the results for an additional “very low efficacy” scenario in Supplementary Methods Sec. 1.3.7 as well as Supplementary Table 6).

Next, we also account for the fact that the infectiousness of children and adolescents has been a matter of debate^24–26,48,49^. While for all analyses presented above we assumed reduced infectiousness for those respective age groups compared to adults and elderly, we now assume (as an upper limit) that children and adolescents are as infectious as adults (see Supplementary Methods, Sec. 1.3.3). This generally leads to higher contributions by unvaccinated individuals to the overall share of infections since they represent by far the majority in these age groups. We find that the unvaccinated in this scenario cause 76%–85% of all new infections for the “medium” and “high” scenario, respectively (see Supplementary Tables 1 and 2) which is substantially larger than the 67%–76% obtained when susceptibility and infectiousness in children and adolescents is reduced (see again Fig. 1a, b and Tabs. 2, 3).

Moreover, we test how our results change if the assumption of homogeneous mixing between vaccinated and unvaccinated individuals is no longer met. This captures the likely scenario that vaccinated and unvaccinated populations are more prone to meet individuals of similar vaccination status rather than opposing vaccination status either due to homophily^50–52^ or deliberate non-pharmaceutical interventions, such as limiting access to public gatherings, immune shielding^53^, or social distancing informed by serological testing^54^. We conceptualize this process by scaling the off-diagonal matrix elements indicating offspring caused by vaccinated infecting unvaccinated individuals and vice versa with a constant factor *m* ∈ [0, 1] such that *m* = 1 refers to our base scenario of homogeneous mixing between the two groups, Fig. 2b and Supplementary Methods Sec. 1.3.4. As expected, we find that the relative contribution to ${{{{{{{\mathcal{R}}}}}}}}$ made by the unvaccinated increases monotonically with decreasing *m* (inset of Fig. 2b). In case the system was, in fact, already in a state of heterogeneous mixing during the observational period, this implies that our main results shown in Fig. 1 present lower bounds of the contribution made by unvaccinated individuals. If mixing was decreased by additional NPIs that reduce contacts between unvaccinated and vaccinated individuals, the absolute value of ${{{{{{{\mathcal{R}}}}}}}}$ decreases with decreasing mixing *m*. This illustrates the efficacy such NPIs would have towards mitigation, assuming that the reduced inter-group contacts are not balanced by increased intra-group contact numbers. In the latter case, an increased number of contacts between unvaccinated individuals could even lead to an increase in ${{{{{{{\mathcal{R}}}}}}}}$, potentially worsening the situation.

Ultimately, we investigate how different the situation would have been if vaccine uptake was higher than 65% in the fall of 2021. To this end, we choose the “medium efficacy” scenario, but increase the respective vaccine uptake for adolescents, adults, and elderly to 90% each, leading to an 80% uptake in the total population, Fig. 2c and Supplementary Methods Sec. 1.3.6. In this case, the effective reproduction number would be lowered to a value of ${{{{{{{\mathcal{R}}}}}}}}=0.86$ instead of ${{{{{{{\mathcal{R}}}}}}}}=1.2$, implying epidemic control. Because more people would be vaccinated, both the relative and absolute contributions of vaccinated individuals to ${{{{{{{\mathcal{R}}}}}}}}$ would increase. Yet, the most important differences to the base scenario of *v* = 65% are the respective reductions of the absolute contributions of unvaccinated individuals, which would decrease from (i) *C*_*u*←*u*_ + *C*_*v*←*u*_ = 0.8 to *C*_*u*←*u*_ + *C*_*v*←*u*_ = 0.37 for infections caused and (ii) from *C*_*u*←*u*_ + *C*_*u*←*v*_ = 0.67 to *C*_*u*←*u*_ + *C*_*u*←*v*_ = 0.3 for becoming infected, both more than halved (see Supplementary Tables 4 and 5). Because unvaccinated infecteds have a much higher probability of suffering from severe disease and being hospitalized, such a reduction can be substantial for relieving an overburdened public health system.

## Discussion

After vaccine rollout programs in Germany have slowed down in late summer, incidences were rising to unprecedented levels in the fall of 2021, with hospitals and ICUs having reached maximum capacity. As about 41% of reported cases aged 12 or above were recorded as breakthrough infections in October 2021, two questions naturally arise: (i) How much were the vaccinated still contributing to the infection dynamics and (ii) how need NPIs to be targeted and calibrated to help achieve temporal epidemic control?

Here, we developed a model-based framework that allows for quantifying the contributions of different infection pathways between and across vaccinated and unvaccinated groups towards the effective reproduction number ${{{{{{{\mathcal{R}}}}}}}}$. Based on this framework and reasonable assumptions regarding vaccine efficacy, we conclude that about 61%–76% percent of the effective reproduction number were caused by unvaccinated individuals, with 32%–51% of its value determined by unvaccinated individuals infecting other unvaccinated individuals. Depending on the assumed efficacy scenario, 34%–50% of the infections are expected to be breakthrough infections. Although these numbers might seem comparatively large at first glance, such results that focus solely on the presence or absence of an infection (not the severity) are to be expected^55^. Our study highlights the importance of analyzing the limited contribution these breakthrough cases make towards the overall infection dynamics, especially in relation to the size of the respective vaccinated/unvaccinated subpopulations. Additionally, such proportions of breakthrough infections are not necessarily indicative of a potential burden to the public health system, as all vaccines against COVID-19 have been reported to substantially reduce the risk of a severe course of the disease^11,16–18^.

We further showed that targeted NPIs that would decrease the transmissibility of unvaccinated individuals by 22%–27% could have suppressed epidemic growth reaching ${{{{{{{\mathcal{R}}}}}}}} < 1$, under the assumption that vaccinated individuals would continue to behave as before, i.e., with no additional NPIs in place for this respective group. Yet, it is questionable how well NPIs can be targeted towards single subpopulations, both for ethical and pragmatic reasons. We found that for NPIs that would affect both unvaccinated and vaccinated individuals, those that reduce the transmissibility of the unvaccinated two to three times as strongly as the vaccinated population would reduce ${{{{{{{\mathcal{R}}}}}}}}$ in the most efficient manner.

Our assumptions regarding vaccine efficacy against transmission (effective transmissibility reduction) were lower than values observed in the Netherlands^52^. Assuming that the efficacy is of larger value would further increase the contributions of unvaccinated individuals towards the infection dynamics. Similarly, if children and adolescents were found to be as susceptible and infectious as adults, the contributions made by the unvaccinated subpopulation would be of larger value as well.

The analyses performed here represent model-based estimations that are limited by data quality and a large number of parameters that have to be estimated based on available empirical results. This includes epidemiological data as well as contact data from the POLYMOD study, which is already over 15 years old and might therefore inaccurately portray the mixing behavior of the German population at the time of writing. A further limiting factor is that the under-ascertainment of breakthrough infections might be larger than accounted for, as vaccinated infecteds experiencing mild symptoms might not be as likely to have their infection reported, thus leading to a potential overestimation of vaccine efficacy. Yet, vaccinated individuals might have increased their contact behavior compared to unvaccinated individuals, a behavorial change that compensates for the vaccine-induced lowered individual risk of infection. Because vaccine efficacies were estimated using Farrington’s method^11,55^, such a relative increase in contact behavior of vaccinated individuals could lead to an underestimation of the true vaccine efficacy, thus potentially balancing a hypothetical inequality in ascertainment. Due to such uncertainties, future empirical studies, e.g. using contact tracing data, will be necessary to confirm or refute our claims.

While we consider population mixing across age groups, we also implicitly assume homogeneous mixing between vaccinated and unvaccinated individuals in our base scenarios. Yet, the intention to vaccinate has been shown to follow rules of social contagion, rendering it likely that vaccinated and unvaccinated individuals mix less across groups^56^. We showed that, in this case, the contribution of unvaccinated individuals to ${{{{{{{\mathcal{R}}}}}}}}$ would be of even larger magnitude. NPIs that reduced contacts between both subpopulations (i.e. reduced mixing) would lead to a decrease in ${{{{{{{\mathcal{R}}}}}}}}$, as long as these reductions are not balanced by an increase in contacts among unvaccinated individuals, in which case ${{{{{{{\mathcal{R}}}}}}}}$ might even increase, highlighting the necessity for well-targeted measures. We want to stress that one should be careful, however, not to overinterpret this result as explicit advice for future NPIs to increase segregation between the vaccinated and unvaccinated. Indeed, other research shows that after measures that restricted access to shopping and leisure activities only for the unvaccinated, societal polarization was high^56^. While this may reduce mixing, it creates other, potentially worse societal problems. Our analysis does not account for any psychological or socio-cultural consequences of such policies or recommendations^57^ and, as always, recommendations should be weighed against potential risks.

Finally, an increased vaccine uptake would increase both the relative and absolute contributions that the vaccinated population makes towards ${{{{{{{\mathcal{R}}}}}}}}$ while similarly decreasing the effective reproduction number’s absolute value, potentially leading to temporary epidemic control under the assumption of unchanged behavior. In light of the slow growth of vaccine uptake in Germany after the summer 2021^22^ and low intention to vaccinate among those that are unvaccinated^41^, such an increase in uptake, however, seems unlikely to be achieved.

We furthermore stress that our results are estimations made for the comparatively short period between October 11, 2021 and November 7, 2021. As vaccine efficacy against infection has been reported to decrease with time, fast and wide-spread booster vaccination is a crucial measure to avoid an increasing reproduction number and a potentially worsening public health crisis. Also, the spread of immune escape variants may change the situation.

In summary, our results suggest that a minority of the population (i.e., the unvaccinated) contributed a substantial part to the infection dynamics, thus making them the primary driver of the public health crisis in Germany during the fourth wave of the COVID-19 pandemic and presumably also in other countries that were experiencing similar dynamics. We also show that this effect can be compensated through targeted NPIs that effectively lower the transmissibility of infected, yet unvaccinated, individuals. Hence, our study further underlines the importance of vaccines as a pharmaceutical intervention regarding epidemic control and highlights the importance of increasing vaccine uptake, e.g. through campaigning or low-threshold offers, wherever possible, in order to achieve efficient and long-term epidemic control and preventing an overload of public health systems.

### Supplementary information

Description of Additional Supplementary Files

Supplementary Information

Supplementary Data 1

Reporting Summary

## Data Availability

Analysis results produced in this study are given in the Supplementary Information and on Zenodo (ref. 58). Source data for the figures are available as Supplementary Data 1 and in the Zenodo repository. Data regarding the count of breakthrough infections and estimated vaccine efficacy during the study period may be found in ref. 11. Population sizes and contact numbers were chosen according to ref. 20, based on data from refs. 18–19.

## Code availability

Code to reproduce the results of this study is available under github.com/benmaier/vaccontrib and on Zenodo^58^.
