# Author Correction: Germany’s fourth COVID-19 wave was mainly driven by the unvaccinated

**DOI:** 10.1038/s43856-024-00432-y

**Published:** 2024-01-12

**Authors:** Benjamin F. Maier, Marc Wiedermann, Angelique Burdinski, Pascal P. Klamser, Mirjam A. Jenny, Cornelia Betsch, Dirk Brockmann

**Affiliations:** 1 Institute for Theoretical Biology and Integrated Research Institute for the Life-Sciences, Humboldt-University of Berlin, Philippstr. 13, 10115 Berlin, Germany; 2 Robert Koch Institute, Nordufer 20, 13353 Berlin, Germany; 3 University of Erfurt, Nordhäuserstr. 63, 99089 Erfurt, Germany; 4 Harding Center for Risk Literacy, University of Potsdam, Virchowstrasse 2-4, 14482 Potsdam, Germany; 5 Max Planck Institute for Human Development, Lentzeallee 94, 14195 Berlin, Germany; 6 Bernhard-Nocht-Institut, Bernhard-Nocht-Straße 74, 20359 Hamburg, Germany

**Keywords:** Viral infection, Computational biology and bioinformatics

Correction to: *Communications Medicine* 10.1038/s43856-022-00176-7, published online 16 September 2022

In the Background section of the Abstract, we would like to clarify that the 41% of recorded new cases mentioned is a proportion of recorded new symptomatic infections, not total new infections. The text read ‘…with about 41% of recorded new cases aged twelve or above being symptomatic breakthrough infections…’. It now reads ‘…with about 41% of recorded new symptomatic cases aged twelve or above being symptomatic breakthrough infections…’.

In the Introduction, we omitted a reference to SurvStat at the end of the following sentence: ‘In the four weeks between Oct 11, 2021, and Nov 7, 2021, Germany’s central public health institute, the Robert Koch Institute (RKI) reported 250,552 new symptomatic infections in individuals with known vaccination status, 90,471 of which were attributed to vaccinated individuals, i.e. 36% were symptomatic breakthrough cases (41% in age groups eligible for vaccination)^11^’. This reference (which was previously reference 26 but is now reference 12) has now been added to explain how the figure of 250,552 was derived.

These corrections have been made in the PDF and HTML versions of the article.

