# Supplementary material for: Germany’s fourth COVID-19 wave was mainly driven by the unvaccinated
Source: Commun Med (Lond). 2022 Sep 16;2:116. doi: 10.1038/s43856-022-00176-7 (PMC9481603; doi:10.1038/s43856-022-00176-7)
Supplement: Supplementary file 1 — Description of Additional Supplementary Files [file 43856_2022_176_MOESM1_ESM.pdf]

Description of Additional Supplementary Files

File Name: Supplementary Data 1

Description: Data to reproduce the figures
