# Supplementary material for: Germany’s fourth COVID-19 wave was mainly driven by the unvaccinated
Source: Commun Med (Lond). 2022 Sep 16;2:116. doi: 10.1038/s43856-022-00176-7 (PMC9481603; doi:10.1038/s43856-022-00176-7)
Supplement: Supplementary file 2 — Supplementary Information [file 43856_2022_176_MOESM2_ESM.pdf]

Supplementary Information: Germany's fourth COVID-19 wave was mainly driven by the unvaccinated

Benjamin F. Maier [a],[1,2] Marc Wiedermann,[1,2] Angelique Burdinski,[1,2]
Pascal P. Klamser,[1,2] Mirjam A. Jenny,[3,4,5,6] Cornelia Betsch,[3,6] and Dirk Brockmann[1]

[1]*Institute for Theoretical Biology and Integrated Research Institute for the Life-Sciences, Humboldt-University of Berlin, Philippstr. 13, 10115 Berlin, Germany*

[2]*Robert Koch Institute, Nordufer 20, 13353 Berlin, Germany*

[3]*University of Erfurt, Nordhäuserstr. 63, 99089 Erfurt, Germany*

[4]*Harding Center for Risk Literacy, University of Potsdam, Virchowstrasse 2-4, 14482 Potsdam, Germany*

[5]*Max Planck Institute for Human Development, Lentzeallee 94, 14195 Berlin, Germany*

[6]*Bernhard-Nocht-Institut, Bernhard-Nocht-Straße 74, 20359 Hamburg, Germany*

[a] e-mail: bfmaier@physik.hu-berlin.de

1. SUPPLEMENTARY METHODS

1.1. The contribution matrix

1.1.1. Contribution matrix for vaccinated populations

Consider a generalized compartmental susceptible-infected-removed model that also tracks vaccinated individuals and breakthrough infections. We assume that the population can be sorted into $M \in \mathbb{N}^+$ groups and that a single individual can be in any of $V \in \mathbb{N}^+$ vaccination states. The ordinary differential equations describing the evolution of an epidemic in this model are given as

$$\begin{aligned}
\partial_t S_i^\Gamma &= -\sum_{\Lambda=1}^{V}\sum_{j=1}^{M}\alpha_{ij}^{\Gamma\Lambda} S_i^\Gamma \frac{I_j^\Lambda}{N_j}\\
\partial_t I_i^\Gamma &= \sum_{\Lambda=1}^{V}\sum_{j=1}^{M}\alpha_{ij}^{\Gamma\Lambda} S_i^\Gamma \frac{I_j^\Lambda}{N_j} - \beta_i^\Gamma I_i^\Gamma\\
\partial_t R_i^\Gamma &= \beta_i^\Gamma I_i^\Gamma .
\end{aligned}$$

Here, S_i^Γ represents the compartment counting susceptibles in population group $1 \le i \le M$ of size N_i that have vaccination status $1 \le \Gamma \le V$ (analogously, I_i^Γ represent the respective (breakthrough) infections, and R_i^Γ the respective removed individuals).

We will assume in the following that the epidemic is contained well enough such that the fraction of infected individuals in population group i is always much lower than the respective remaining susceptibles in this group, which means that we can linearize the equations above around the disease-free state. We further argue that outbreaks will quickly push the prevalence into the eigenvector corresponding to the largest eigenvalue of the system's Jacobian. The disease-free state is, for each population group i, given as

$$\begin{aligned}
\sum_{\Gamma=1}^{\Lambda} \tilde{S}_i^\Gamma &= N_i\\
\tilde{I}_i^\Gamma = \tilde{R}_i^\Gamma &= 0,\ \ \forall\Gamma.
\end{aligned}$$

For simplicity, we will assume that $\Gamma = 1$ refers to unvaccinated individuals and $\Gamma > 1$ refers to vaccinated individuals. For example if we consider $V = 2$ in an otherwise homogeneous population, and assume that 60% of a population of size $N = 10^7$ are vaccinated, we have the disease-free state $\tilde{S}^{\Gamma=1} = 4\times 10^6$, $\tilde{S}^{\Gamma=2} = 6\times 10^6$ and $\tilde{I}^\Gamma = \tilde{R}^\Gamma = 0$.

We want to derive the contributions vaccinated individuals make to the effective reproduction number of this system in the regime of small outbreaks. To this end, we use the next-generation-matrix framework[1,2]. Note that we are only interested in contributions to the reproduction number, which remains unchanged by the introduction of latent compartments which we can therefore safely ignore without invalidating our results.

The system's next generation matrix is derived as follows. Consider the transmission matrix of small domain

$$T_{ij}^{\Gamma\Lambda} = \alpha_{ij}^{\Gamma\Lambda}\tilde{S}_i^\Gamma N_j^{-1}$$

and the transition matrix of small domain

$$\Sigma_{ij}^{\Gamma\Lambda} = \beta_i^\Gamma \delta_{ij}\delta_{\Gamma\Lambda}$$

with Kronecker's symbol

$$\delta_{ij} = \begin{cases} 1 & \text{if } i = j, \\ 0 & \text{otherwise.} \end{cases}$$

Note that both of these matrices are actually tensors of size $M \times M \times V \times V$ and capture the reproduction dynamics caused by infecteds of group j and vaccination status Λ towards susceptibles of group i and of vaccination status Γ.

Now, the next generation matrix $\boldsymbol{K}$ is given as

$$\boldsymbol{K} = \boldsymbol{T}\Sigma^{-1}.$$

Since Σ is diagonal, we can write down $\boldsymbol{K}$ explicitly, namely as

$$K_{ij}^{\Gamma\Lambda} = \frac{\alpha_{ij}^{\Gamma\Lambda} \tilde{S}_i^{\Gamma}}{\beta_j^{\Lambda} N_j}.$$

The reproduction number of this system is given as the spectral radius of the next generation matrix

$$\mathcal{R} = \rho(\boldsymbol{K}).$$

Around the disease-free equilibrium, the system can be linearized. Consider a vector $\boldsymbol{y}$ whose elements y_i^{Γ} contains the number of newly infecteds in population group i and vaccination status Γ. The per-generation growth of these infecteds will effectively follow

$$\boldsymbol{y}(g+1) = \boldsymbol{K}\boldsymbol{y}(g),$$

which means the incidence $\boldsymbol{y}$ will quickly approach a state that points in the direction of the eigenvector $\hat{\boldsymbol{y}}$ corresponding to the largest eigenvalue $\mathcal{R}$. Let's normalize this eigenvector such that

$$\sum_{\Gamma=1}^{V} \sum_{i=1}^{M} \hat{y}_i^{\Gamma} = 1.$$

With

$$\boldsymbol{K}\hat{\boldsymbol{y}} = \mathcal{R}\hat{\boldsymbol{y}},$$

this implies

$$\sum_{\Gamma=1}^{V} \sum_{i=1}^{M} \left(\sum_{\Lambda=1}^{V} \sum_{j=1}^{M} K_{ij}^{\Gamma\Lambda} \hat{y}_j^{\Lambda} \right) = \mathcal{R}.$$

From this equation, we can read off the contribution matrix

$$\begin{aligned} C_{ij}^{\Gamma\Lambda} &= K_{ij}^{\Gamma\Lambda} \hat{y}_j^{\Lambda} \\ &= \frac{\alpha_{ij}^{\Gamma\Lambda} \tilde{S}_i^{\Gamma}}{\beta_j^{\Lambda} N_j} \times \hat{y}_j^{\Lambda}, \end{aligned} \tag{S1}$$

which contains the contributions to $\mathcal{R}$ made by infected individuals of group j and vaccination status Λ towards susceptibles of group i and vaccination status Γ in the regime of small outbreaks. To obtain average population-wide contributions of vaccinated and unvaccinated individuals, we can simply sum over the contributions of all population groups to find

$$C^{\Gamma\Lambda} = \sum_{i=1}^{M} \sum_{j=1}^{M} C_{ij}^{\Gamma\Lambda}. \tag{S2}$$

1.1.2. Constructing the next generation matrix in presence of vaccination

In this section we want to introduce some quantities that clarify the construction of the infection rate matrix $\boldsymbol{\alpha}$, the next generation matrix $\boldsymbol{K}$, and how to simplify the approach.

Let's begin with the base case, i. e. the absence of working vaccines in which $V = 1$ (we only have one vaccination status which is "unvaccinated"). We further introduce α_j as the vector quantifying population group-specific infection rates because viral shedding could be, for instance, age-specific, as well as the contact matrix γ_{ij} that contains the "typical" number of contacts a randomly chosen individual of group j has towards individuals of group i. This implies

$$\alpha_{ij} = \gamma_{ij}\alpha_j$$

and

$$K_{ij} = \gamma_{ij}\alpha_j \frac{N_i}{N_j}\frac{1}{\beta_j},$$

i. e.

$$\boldsymbol{K} = \mathrm{diag}(\boldsymbol{N}) \cdot \boldsymbol{\gamma} \cdot \mathrm{diag}(\boldsymbol{N})^{-1} \cdot \mathrm{diag}(\boldsymbol{\alpha}) \cdot \mathrm{diag}(\boldsymbol{\beta})^{-1}.$$

Here, β_j^{-1} quantifies the average duration of the infectious period of an individual of group j.

Now, let's assume that we do not know the explicit viral shedding rate (or infectiousness), but that we can make reasonable assumptions about their relative size (e.g. "individuals of group j are twice as infectious as individuals of group i" which would imply $\alpha_j/\alpha_i = 2$), such that

$$\alpha_j = \alpha_0 a_j$$

with $\boldsymbol{a}$ being a vector that contains these relative values. Likewise, we can scale the relative duration of the average infectious periods with

$$\beta_j = \beta_0 b_j.$$

What we usually do have estimates for is the basic reproduction number, which we find as

$$\begin{aligned}
\mathcal{R}_0 &= \rho(\boldsymbol{K}) \\
&= \alpha_0\beta_0\rho\left(\mathrm{diag}(\boldsymbol{N}) \cdot \boldsymbol{\gamma} \cdot \mathrm{diag}(\boldsymbol{N})^{-1} \cdot \mathrm{diag}(\boldsymbol{a}) \cdot \mathrm{diag}(\boldsymbol{b})^{-1}\right) \\
&= \alpha_0\beta_0\rho\left(\boldsymbol{\gamma} \cdot \mathrm{diag}(\boldsymbol{a}) \cdot \mathrm{diag}(\boldsymbol{b})^{-1}\right) \\
&= \alpha_0\beta_0\rho_0
\end{aligned}$$

with

$$\rho_0 = \rho\left(\boldsymbol{\gamma} \cdot \mathrm{diag}(\boldsymbol{a}) \cdot \mathrm{diag}(\boldsymbol{b})^{-1}\right).$$

Above, we used the fact that diagonal operators commute and that the spectrum of matrices remains unchanged under base transformations. With this result we can gauge α_0 as

$$\alpha_0 = \mathcal{R}_0\beta_0\rho_0^{-1}.$$

Now, we introduce vaccines. Let's assume that vaccinated individuals of vaccination status Λ in population group j have status-dependent transmissibility reduction of

$$0 \le r_j^{\Lambda} \le 1$$

when they suffer from a breakthrough infection. In the following we will assume that $\Lambda = 1$ corresponds to unvaccinated individuals such that $r_j^{\Lambda=1} = 0$. Likewise, we introduce a susceptibility reduction for individuals of population group i and vaccination status Γ of

$$0 \leq s_i^{\Gamma} \leq 1$$

where, again, we assume that $s_i^{\Gamma=1} = 0$. We therefore define as the infection rate matrix

$$\alpha_{ij}^{\Gamma\Lambda} = \frac{\mathcal{R}_0 \beta_0}{\rho_0} \gamma_{ij} (1 - s_i^{\Gamma})(1 - r_j^{\Lambda}) a_j$$

such that the next generation matrix is given as

$$K_{ij}^{\Gamma\Lambda} = \frac{\mathcal{R}_0}{\rho_0} \gamma_{ij} (1 - s_i^{\Gamma})(1 - r_j^{\Lambda}) \frac{\tilde{S}_i^{\Gamma} a_j}{N_j b_j^{\Lambda}}.$$

With $(b_j^{\Lambda})^{-1}$ we acknowledge that the average infectious period can differ for vaccinated individuals. This result is independent of exact values for infection rates α_0 and average infectious period β_0^{-1}.

We can further encapsulate restrictions imposed on (un)vaccinated individuals by making the "basic" reproduction number depend on vaccination status of infectious individuals such that

$$K_{ij}^{\Gamma\Lambda} = \frac{\mathcal{R}_0^{\Lambda}}{\rho_0} \gamma_{ij} (1 - s_i^{\Gamma})(1 - r_j^{\Lambda}) \frac{\tilde{S}_i^{\Gamma} a_j}{N_j b_j^{\Lambda}}.$$

Regarding mixing behavior, we can introduce an unvaccinated-vaccinated mixing matrix that mimics social segregation of vaccinated and unvaccinated individuals. For simplicity, we will assume that this mixing matrix is independent of the size of the respective subpopulations and given as

$$\mu = \begin{pmatrix} 1 & m & m & \dots & m \\ m & 1 & 1 & \dots & 1 \\ m & 1 & 1 & \dots & 1 \\ \vdots & \vdots & \vdots & \ddots & \vdots \\ m & 1 & 1 & \dots & 1 \end{pmatrix}$$

with $0 < m \leq 1$. Then, the next-generation matrix is given as

$$K_{ij}^{\Gamma\Lambda} = \frac{\mathcal{R}_0^{\Lambda}}{\rho_0} \gamma_{ij} \mu^{\Gamma\Lambda} (1 - s_i^{\Gamma})(1 - r_j^{\Lambda}) \frac{\tilde{S}_i^{\Gamma} a_j}{N_j b_j^{\Lambda}}. \qquad \text{(S3)}$$

Hence, the parameter m controls the amount of mixing between unvaccinated and vaccinated individuals with $m = 1$ yielding homogeneous mixing and $m < 1$ implying less mixing between unvaccinated and vaccinated states.

Note that if any $s_i^{\text{unvacc}} \neq 1$ or $r_i^{\text{unvacc}} \neq 1$, ρ_0 has to be computed as

$$\rho_0 = \rho \left(\text{diag}(1 - \boldsymbol{s}^0) \cdot \boldsymbol{\gamma} \cdot \text{diag}(\boldsymbol{a}) \cdot \text{diag}(\boldsymbol{b}^0)^{-1} \cdot \text{diag}(1 - \boldsymbol{r}^0) \right)$$

where $s_i^0 = s_i^{\text{unvacc}}$, $r_i^0 = r_i^{\text{unvacc}}$ and $b_i^0 = b_i^{\text{unvacc}}$. Once $K_{ij}^{\Lambda\Gamma}$ has been constructed, we compute its spectral radius and corresponding normalized eigenvector $\hat{y}_j^{\Gamma}$ and both the full (Eq. (S1)) and the reduced contribution matrix Eq. (S2).

1.1.3. Explicit derivation of the contribution matrix in a homogeneous population

We devise a simplified susceptible-infected-removed model[3], where both (unvaccinated) susceptible (S) and vaccinated (V) individuals can be infected by the unvaccinated (I) and vaccinated infectious (Y) population. Both recover to either unvaccinated recovered, (R), or vaccinated recovered (X).

For simplicity, we assume full immunity after recovery, which means that the duration of immunity is much longer than the time scale at which new outbreaks occur. Each variable reflects the relative frequency of the respective individuals in a population of size N, implying that $S+I+R+V+Y+X=1$. Accounting for potentially different infection rates α_Y and α_I of vaccinated and unvaccinated, susceptibles are depleted in a well-mixed system as

$$\frac{dS}{dt} = -(\alpha_I I + \alpha_Y Y)S. \tag{S4}$$

Additionally, we denote the total prevalence as $\mathcal{I} = I + Y$. In addition, not explicitly discriminating vaccinated and unvaccinated infected individuals yields

$$\frac{dS}{dt} = -\alpha_S \mathcal{I} S = -\beta \tilde{\mathcal{R}}_S \mathcal{I} S = -\beta \mathcal{R}_S \mathcal{I}. \tag{S5}$$

Here, $\tilde{\mathcal{R}}_S = \alpha_S/\beta$ is the *basic* reproduction number that represents the typical number of offspring per typical infectious individual in a fully susceptible population and β is a generalized recovery rate. In addition, $\mathcal{R}_S$ is the *effective* reproduction number that represents the number of offspring per typical individual in the remaining susceptible population, i.e., $\mathcal{R}_S = \tilde{\mathcal{R}}_S S$. Combining Eq. (S4) and Eq. (S5) then yields a closed formula for $\mathcal{R}_S$ as

$$\mathcal{R}_S = \frac{(\alpha_I I + \alpha_Y Y)S}{\beta \mathcal{I}} \tag{S6}$$

Note, that an analogous derivation can be done for the share of vaccinated individuals V, which yields a basic reproduction number $\mathcal{R}_V$ such that the total effective reproduction number reads $\mathcal{R} = \mathcal{R}_S + \mathcal{R}_V$.

The main goal of pandemic control is to decrease $\mathcal{R}$, e.g., by vaccination campaigns. Assuming a vaccine efficacy of $0 \le s \le 1$ against infection and a vaccine uptake $0 \le v \le 1$ homogeneously distributed over the whole population, the total number of unvaccinated individuals at risk of infection is given as $S = 1 - v$ and the total number of vaccinated individuals at risk of infection is given as $V = v(1-s)$, such that $S + V = 1 - vs$.

For low prevalence, i.e., $\mathcal{I} \ll 1$, an infectious individual will infect vaccinated and unvaccinated proportionally, such that the probability for a randomly chosen infected to have come from the unvaccinated susceptible population reads $p_I = (1-v)/(1-vs)$. Likewise, the probability for a randomly chosen infected to originate from the vaccinated population reads $p_Y = v(1-s)/(1-vs)$. We can therefore explicitly disentangle the total infectious population $\mathcal{I}$ into its two contributions

$$I = \frac{1-v}{1-vs}\mathcal{I} \quad \text{and} \quad Y = \frac{v(1-s)}{1-vs}\mathcal{I}. \tag{S7}$$

One principle question of this study is how infectious the vaccinated population will be towards the unvaccinated population if their respective base transmissibilities are reduced by targeted non-pharmaceutical interventions (NPIs). Hence, we set $\alpha_Y = \alpha_v(1-r)$. Here, r represents the reduction of transmissibility per contact that arises from vaccination and α_v represents the base transmissibility of vaccinated individuals under targeted NPIs (e.g. contact reductions). In contrast,

unvaccinated individuals will transmit pathogens with a transmission rate $\alpha_I = \alpha_u$, with α_u representing a reduced base transmissibility caused by NPIs that target the unvaccinated population. Plugging these assumptions as well as Eq. (S7) into Eq. (S6) yields

$$\begin{aligned}\mathcal{R}_S &= \frac{[\alpha_u(1-v)\mathcal{I} + \alpha_v(1-r)v(1-s)\mathcal{I}](1-v)}{\beta\mathcal{I}(1-vs)} \\ &= \frac{1-v}{1-vs}[\mathcal{R}_u(1-v) + \mathcal{R}_v v(1-r)(1-s)].\end{aligned}$$

$\mathcal{R}$ can thus be decomposed into the respective contributions by vaccinated and unvaccinated individuals such that

$$\begin{aligned}\mathcal{R}_{I\to S} &= \frac{(1-v)^2}{1-vs}\mathcal{R}_u \\ \mathcal{R}_{Y\to S} &= \frac{v(1-v)(1-s)(1-r)}{1-vs}\mathcal{R}_v\end{aligned}$$

Analogously, we find the contributions of vaccinated and unvaccinated individuals towards the infection of vaccinated individuals as

$$\begin{aligned}\mathcal{R}_{I\to V} &= \frac{v(1-v)(1-s)}{1-vs}\mathcal{R}_u \\ \mathcal{R}_{Y\to V} &= \frac{v^2(1-s)^2(1-r)}{1-vs}\mathcal{R}_v\end{aligned}$$

Plugging all four contributions together yields the total effective reproduction number

$$\begin{aligned}\mathcal{R} &= \mathcal{R}_{I\to S} + \mathcal{R}_{Y\to S} + \mathcal{R}_{I\to V} + \mathcal{R}_{Y\to V} \\ &= (1-v)\mathcal{R}_u + v(1-s)(1-r)\mathcal{R}_v. \end{aligned} \tag{S8}$$

The contribution matrix is given as

$$\boldsymbol{C} = \begin{pmatrix}\mathcal{R}_{I\to S} & \mathcal{R}_{Y\to S} \\ \mathcal{R}_{I\to V} & \mathcal{R}_{Y\to V}\end{pmatrix}. \tag{S9}$$

1.1.4. Operational definition based on a two-dimensional example

Consider M coupled populations, individuals of which produce new individuals in each of these populations. A next generation matrix K_{ij} of shape $M \times M$ contains the average offspring a single j individual produces in population i.

For instance, the matrix

$$\boldsymbol{K} = \begin{pmatrix}1 & 1 \\ 2 & 3\end{pmatrix} \tag{S10}$$

describes a system of two populations, let's call them A and B with indices $i_A = 0$ and $i_B = 1$. In one generation (i. e. during its lifetime), a single A individual produces, on average, $K_{00} = 1$ individuals in population A and $K_{10} = 2$ individuals in population B. A single B individual produces, on average, $K_{11} = 3$ individuals in population A and $K_{01} = 1$ individuals in population A.

Let the vector $\boldsymbol{y}(g)$ of length $M = 2$ contain the number of A- and B-individuals, respectively, at generation g. The per-generation dynamics follow

$$\boldsymbol{y}(g+1) = \boldsymbol{K}\boldsymbol{y}(g).$$

After a few generations, the system state $\boldsymbol{y}$ approaches the eigenvector of $\boldsymbol{K}$ that corresponds to its largest eigenvalue (spectral radius). We can compute the relative size of populations A and B as

$$\hat{\boldsymbol{y}} = \begin{pmatrix} A \\ B \end{pmatrix} = \begin{pmatrix} 0.27 \\ 0.73 \end{pmatrix}.$$

Now, we want to define the so-called "contribution matrix" which quantifies the absolute contributions of each population to the reproduction of each respective population when the exponential growth (or decay) has approached the eigenstate.

Operationally, one can define the contribution matrix as follows. During a time of growth (decay), we track newborn individuals of both populations A and B for a few generations. Let's call the set of these individuals $\mathcal{I}$. For each individual $i \in \mathcal{I}$, we track the count of its offspring in the respective populations A and B. Let's define as

$$\underline{A}(i) = \begin{cases} 1 & \text{if } i \text{ belonged to } A, \\ 0 & \text{otherwise} \end{cases}$$

and

$$\underline{B}(i) = \begin{cases} 1 & \text{if } i \text{ belonged to } B, \\ 0 & \text{otherwise} \end{cases}$$

functions that give information about the populations individuals $i \in \mathcal{I}$ belonged to. Hence, $\mathcal{I}_A = \{i : i \in \mathcal{I} \wedge \underline{A}(i) = 1\}$ and $\mathcal{I}_B = \{i : i \in \mathcal{I} \wedge \underline{B}(i) = 1\}$ are the respective subsets of $\mathcal{I}$ that contain A and B individuals, respectively.

We further define as $\sigma_p(i)$ the number of p-offspring that individual i produced during its lifetime. Then we can define the offspring matrix

$$\boldsymbol{P} = \sum_{i \in \mathcal{I}} \begin{pmatrix} \sigma_A(i)\underline{A}(i) & \sigma_A(i)\underline{B}(i) \\ \sigma_B(i)\underline{A}(i) & \sigma_B(i)\underline{B}(i) \end{pmatrix}$$

whose entries P_{ij} quantify how much i-offspring has been produced by j-individuals during the measurement period. Given the definitions of the sets above, we can also write $\boldsymbol{P}$ as

$$\boldsymbol{P} = \begin{pmatrix} \sum_{i \in I_A} \sigma_A(i) & \sum_{i \in I_B} \sigma_A(i) \\ \sum_{i \in I_A} \sigma_B(i) & \sum_{i \in I_B} \sigma_B(i) \end{pmatrix}.$$

The relative contribution matrix is then defined as

$$\tilde{\boldsymbol{C}} = \frac{P}{\sum_{i \in \mathcal{I}} \left(\sigma_A(i) + \sigma_B(i)\right)}.$$

Each entry $\tilde{C}_{ij}$ contains the j-produced number of i-offspring relative to the total number of offspring in the system during the measurement period.

The average number of offspring per any individual is given as

$$\mathcal{R} = \frac{1}{|\mathcal{I}|} \sum_{i \in \mathcal{I}} \Big(\sigma_A(i) + \sigma_B(i) \Big).$$

This number is also called the "basic reproduction number" because it quantifies the average number of offspring per "typical" infectious individual. So in order to find the absolute contributions of j-induced i-offspring to the reproduction number we define the contribution matrix

$$\boldsymbol{C} = \mathcal{R} \tilde{\boldsymbol{C}},$$

which evaluates to

$$\boldsymbol{C} = \frac{1}{|\mathcal{I}|} \begin{pmatrix} \sum_{i \in I_A} \sigma_A(i) & \sum_{i \in I_B} \sigma_A(i) \\ \sum_{i \in I_A} \sigma_B(i) & \sum_{i \in I_B} \sigma_B(i) \end{pmatrix}.$$

We can also define the next generation matrix operationally. First, be reminded that $\mathcal{I}_A$ and $\mathcal{I}_B$ are the respective subsets of $\mathcal{I}$ that contain A and B individuals, respectively. Then

$$\hat{\boldsymbol{y}} = \frac{1}{|\mathcal{I}|} \begin{pmatrix} |\mathcal{I}_A| \\ |\mathcal{I}_B| \end{pmatrix}$$

describes the state of the system in terms of newly generated individuals. To find $\boldsymbol{K}$ we want to obtain the average number of i-offspring per active j individual, i. e.

$$\boldsymbol{K} = \begin{pmatrix} \frac{1}{|\mathcal{I}_A|} \sum_{i \in I_A} \sigma_A(i) & \frac{1}{|\mathcal{I}_B|} \sum_{i \in I_B} \sigma_A(i) \\ \frac{1}{|\mathcal{I}_A|} \sum_{i \in I_A} \sigma_B(i) & \frac{1}{|\mathcal{I}_B|} \sum_{i \in I_B} \sigma_B(i) \end{pmatrix}.$$

We then see that

$$\boldsymbol{K} \cdot \mathrm{diag}(\hat{\boldsymbol{y}}) = \begin{pmatrix} \frac{1}{|\mathcal{I}|} \sum_{i \in I_A} \sigma_A(i) & \frac{1}{|\mathcal{I}|} \sum_{i \in I_B} \sigma_A(i) \\ \frac{1}{|\mathcal{I}|} \sum_{i \in I_A} \sigma_B(i) & \frac{1}{|\mathcal{I}|} \sum_{i \in I_B} \sigma_B(i) \end{pmatrix} \tag{S11}$$

$$= \frac{1}{|\mathcal{I}|} \begin{pmatrix} \sum_{i \in I_A} \sigma_A(i) & \sum_{i \in I_B} \sigma_A(i) \\ \sum_{i \in I_A} \sigma_B(i) & \sum_{i \in I_B} \sigma_B(i) \end{pmatrix} \tag{S12}$$

so

$$\boldsymbol{K} \cdot \mathrm{diag}(\hat{\boldsymbol{y}}) = \boldsymbol{C}.$$

Note that the difference between $\boldsymbol{K}$ and $\boldsymbol{C}$ is subtle but important: While K_{ij} contains the average number of i-offspring by a single j-individual, C_{ij} quantifies the average number of j-caused i-offspring per individual, i. e. makes the important distinction to consider the relative amount of j-individuals in $\mathcal{I}$.

For our toy model, we can therefore easily quantify the contribution matrix by computing the eigenvector of the next generation matrix and plugging it into the equation above,

$$\boldsymbol{C} = \begin{pmatrix} 0.27 & 0.73 \\ 0.54 & 2.20 \end{pmatrix}.$$

Here we see that by far the largest contribution to the reproduction number is by B individuals that produce other B individuals.

Note that this does not necessarily mean that, if we wanted to stifle growth altogether to induce decay, it would be enough to hinder B-individuals from reproducing. Let's say that we somehow manage to stop B-individuals from reproducing altogether, such that only A-individuals can produce offspring (either A or B). This means that the next generation matrix is modified as

$$\boldsymbol{K}^* = \begin{pmatrix} 1 & 0 \\ 2 & 0 \end{pmatrix} \tag{S13}$$

which means that the contribution matrix changes to

$$\boldsymbol{C}^* = \begin{pmatrix} 1/3 & 0 \\ 2/3 & 0 \end{pmatrix} \tag{S14}$$

with $\mathcal{R} = 1$. So the population will stay constant over time. Note that the respective absolute contributions by population A in $\boldsymbol{C}^*$ are now of greater value than those in $\boldsymbol{C}$.

1.2. Parameters and scenarios

We report here the matrices and vectors used in the analyses in the main text, constructed based on the values and estimates reported in the Methods section.

1.2.1. Population and contact data

As described in the Methods section, we consider $M = 4$ subpopulations of size

$$\boldsymbol{N} = \begin{pmatrix} 9,137,232 \\ 5,339,517 \\ 46,495,023 \\ 20,275,029 \end{pmatrix},$$

with the contact matrix

$$\boldsymbol{\gamma} = \begin{pmatrix} 2.8394495 & 0.5205262 & 3.235192 & 0.6269835 \\ 0.8907488 & 4.4044118 & 4.745159 & 0.4811966 \\ 0.6357820 & 0.5449370 & 6.430791 & 1.0125184 \\ 0.2825591 & 0.1267252 & 2.321924 & 2.1267606 \end{pmatrix},$$

as constructed using the socialmixr software package[4] based on the POLYMOD (2005) data[5].

1.2.2. Base epidemiological parameters

As argued in the Methods section, we assume that children and adolescents have lower viral shedding rates if infected and set

$$\boldsymbol{a} = \begin{pmatrix} 0.63 \\ 0.81 \\ 1.00 \\ 1.00 \end{pmatrix}.$$

Regarding relative recovery rates, we assume that the infectious period of breakthrough infections is, on average, only 2/3 as long as the infectious period of unvaccinated infecteds, such that

$$\boldsymbol{b} = \begin{pmatrix} 1 & 1.5 \\ 1 & 1.5 \\ 1 & 1.5 \\ 1 & 1.5 \end{pmatrix},$$

In consistence with the average fraction of fully vaccinated individuals, we define the disease-free state as

$$\tilde{\boldsymbol{S}} = \begin{pmatrix} N_1 & 0 \\ (1-0.401)N_2 & 0.401 \times N_2 \\ (1-0.724)N_3 & 0.724 \times N_3 \\ (1-0.851)N_4 & 0.851 \times N_4 \end{pmatrix}.$$

We also assume homogeneous mixing between vaccinated and unvaccinated

$$\boldsymbol{\mu} = \begin{pmatrix} 1 & 1 \\ 1 & 1 \end{pmatrix},$$

a homogeneous vaccine-induced transmissibility reduction of

$$\boldsymbol{r} = \begin{pmatrix} 0 & 0.1 \\ 0 & 0.1 \\ 0 & 0.1 \\ 0 & 0.1 \end{pmatrix},$$

and equal base transmissibility $\mathcal{R}^u = \mathcal{R}^v$.

1.2.3. Scenario “low efficacy”

Regarding the age-dependent susceptibility reduction we set

$$\boldsymbol{s} = \begin{pmatrix} 0.28 & 0.64 \\ 0.28 & 0.64 \\ 0.00 & 0.50 \\ 0.00 & 0.40 \end{pmatrix}.$$

Note that here, a reduced base susceptibility was assumed for children and adolescents ($1-0.28 = 1-s = 72\%$ of the value of adults). Setting a vaccine efficacy of 50% therefore amounts to a total susceptibility reduction that is $1-(1-0.28)\times(1-0.60) = 0.64$ (in relation to full susceptibility associated with adults).

1.2.4. Scenario “medium efficacy”

Regarding the age-dependent susceptibility reduction we set

$$\boldsymbol{s} = \begin{pmatrix} 0.28 & 0.712 \\ 0.28 & 0.712 \\ 0.00 & 0.600 \\ 0.00 & 0.500 \end{pmatrix}.$$

Note that here, a reduced base susceptibility was assumed for children and adolescents ($1-0.28 = 1-s = 72\%$ of the value of adults). Setting a vaccine efficacy of 60% therefore amounts to a total susceptibility reduction that is $1-(1-0.28)\times(1-0.60) = 0.712$ (in relation to full susceptibility associated with adults).

1.2.5. Scenario "high efficacy"

Regarding the age-dependent susceptibility reduction we set

$$\boldsymbol{s} = \begin{pmatrix} 0.28 & 0.9424 \\ 0.28 & 0.9424 \\ 0.00 & 0.7200 \\ 0.00 & 0.7200 \end{pmatrix}.$$

Note that here, a reduced base susceptibility was assumed for children and adolescents ($1-0.28 = 1-s = 72\%$ of the value of adults). Setting a vaccine efficacy of 92% therefore amounts to a total susceptibility reduction that is $1-(1-0.28)\times(1-0.92) = 0.9424$ (in relation to full susceptibility associated with adults).

1.3. Analyses

1.3.1. Systematically decreasing vaccine efficacy

We use Eq. (S3) and assume an age-independent susceptibility reduction $0 \le \sigma \le 1$, such that

$$\boldsymbol{s} = \begin{pmatrix} 0.28 & 1-(1-0.28)\times(1-\sigma) \\ 0.28 & 1-(1-0.28)\times(1-\sigma) \\ 0.00 & \sigma \\ 0.00 & \sigma \end{pmatrix}. \tag{S15}$$

In an "optimistic" scenario, $\boldsymbol{r}$ and $\boldsymbol{b}$ remain constant as defined above. In a "pessimistic" scenario, we assume that $\boldsymbol{r}$ and $\boldsymbol{b}$ are reduced proportionally to σ with

$$\boldsymbol{b} = \begin{pmatrix} 1 & \sigma/2+1 \\ 1 & \sigma/2+1 \\ 1 & \sigma/2+1 \\ 1 & \sigma/2+1 \end{pmatrix}, \tag{S16}$$

and

$$\boldsymbol{r} = \begin{pmatrix} 0 & 0.1\sigma \\ 0 & 0.1\sigma \\ 0 & 0.1\sigma \\ 0 & 0.1\sigma \end{pmatrix}. \tag{S17}$$

1.3.2. *Decreasing the transmissibility of unvaccinated and unvaccinated with targeted NPIs*

In order to obtain Fig. 3a in the main text, we use the "low efficacy", "medium efficacy", and "high efficacy" scenario parameters and gauge the initial base transmissibilities as $\mathcal{R}_0^u = \mathcal{R}_0^v$, such that the spectral radius of $K_{ij}^{\Lambda\Gamma}$ is equal to $\mathcal{R} = 1.2$. Then, we linearly scale $\mathcal{R}^u$ from $\zeta_u = 1 - \mathcal{R}^u/\mathcal{R}_0^u = 0$ to $\zeta_u = 1 - \mathcal{R}^u/\mathcal{R}_0^u = 1/2$, numerically finding the value $\zeta_v = 1 - \mathcal{R}^v/\mathcal{R}_0^v$ at which the spectral radius becomes $\mathcal{R} = 1$.

The resulting isoclines marking $\mathcal{R} = 1$ are linear functions. In the homogeneous cases, we can use Eq. (S8) to find the parametric equation

$$A = (1-v)(1-\zeta_u) + v(1-s)(1-r)(1-\zeta_v)$$

where A is a constant. We rewrite the equation above as

$$\tilde{A} = (1-v)\zeta_u + v(1-s)(1-r)\zeta_v,$$

giving

$$\zeta_v = \tilde{A}' - \frac{1-v}{v(1-s)(1-r)}\zeta_u$$

which determines the isoclines. The linear function that runs perpendicular to this function has slope

$$\chi = \frac{v(1-s)(1-r)}{1-v}$$

and is the "fastest" way to reach any isocline in the plane from any point in the plane.

1.3.3. *Assuming children are as susceptible and infectious as adults*

Here, we assume

$$\boldsymbol{a} = \begin{pmatrix} 1 \\ 1 \\ 1 \\ 1 \end{pmatrix},$$

as well as

$$\boldsymbol{s} = \begin{pmatrix} 0.00 & 0.60 \\ 0.00 & 0.60 \\ 0.00 & 0.60 \\ 0.00 & 0.50 \end{pmatrix}$$

for the "medium efficacy" and

$$\boldsymbol{s} = \begin{pmatrix} 0.00 & 0.92 \\ 0.00 & 0.92 \\ 0.00 & 0.72 \\ 0.00 & 0.72 \end{pmatrix}$$

for the "high efficacy" scenario, respectively. The results are presented in Supplementary Table 1 and Supplementary Table 2.

1.3.4. Decreasing mixing between vaccinated and unvaccinated

One may assume that the intention to vaccinate follows the rules of social contagion, such that it is likely that vaccinated and unvaccinated individuals meet each other less often than they meet individuals with whom they share their respective vaccination status. We can simulate such a hypothetical scenario using Eq. (S3) with

$$\boldsymbol{\mu} = \begin{pmatrix} 1 & m \\ m & 1 \end{pmatrix}.$$

We find that decreasing mixing (decreasing m from $m = 1$ to lower values $0 \leq m < 1$) between vaccinated and unvaccinated individuals decreases $\mathcal{R}$, but increases the relative contributions unvaccinated individuals make towards it (see Fig. 3b in the main text).

1.3.5. Assuming lower vaccine efficacy for the elderly

We base the following scenario on the "medium efficacy" scenario and additionally assume that the elderly have lower protection against infection by setting

$$\boldsymbol{s} = \begin{pmatrix} 0.00 & 0.60 \\ 0.00 & 0.60 \\ 0.00 & 0.60 \\ 0.00 & 0.40 \end{pmatrix},$$

$$\boldsymbol{r} = \begin{pmatrix} 0.00 & 0.10 \\ 0.00 & 0.10 \\ 0.00 & 0.10 \\ 0.00 & 0.08 \end{pmatrix},$$

and

$$\boldsymbol{b} = \begin{pmatrix} 1 & 1.5 \\ 1 & 1.5 \\ 1 & 1.5 \\ 1 & 1.4 \end{pmatrix},$$

effectively reducing all relevant quantities regarding vaccine efficacies in the elderly by 20% (relative to the base value), i.e. setting $\sigma = 0.8$ in the respective last rows of Eqs. (S15)-(S17) and $\sigma = 1$ in the remaining rows. The results are shown in Supplementary Table 3. Comparing these results with those obtained in the "medium efficacy" scenario (Table 3 in the main text) shows that this medium efficacy does not change the original results substantially.

1.3.6. Increasing vaccine uptake

We use the "medium efficacy" scenario and replace the disease-free state with

$$\tilde{\boldsymbol{S}} = \begin{pmatrix} N_1 & 0 \\ (1-0.9)N_2 & 0.9\times N_2 \\ (1-0.9)N_3 & 0.9\times N_3 \\ (1-0.9)N_4 & 0.9\times N_4 \end{pmatrix}.$$

The absolute contributions that are shown in Fig. 3c of the main text are reported in Supplementary Tables 4,5.

1.3.7. Scenario "very low efficacy"

Additionally, we test how this result would change if the effective transmission reduction r' decreases to $r' = 20\%$ for the elderly and adults, keeping $r' = 40\%$ for adolescents, who have not been eligible to receive a vaccine until shortly before October 2021. We obtain Supplementary Table 6.

In this case, all entries in the contribution matrix are relatively balanced, with the unvaccinated still contributing more to the total dynamics than the vaccinated. The largest single entry in the contribution matrix is still given by unvaccinated infecting other unvaccinated.

SUPPLEMENTARY REFERENCES

[1] Diekmann, O., Heesterbeek, J. A. P. & Roberts, M. G. The construction of next-generation matrices for compartmental epidemic models. *Journal of the Royal Society, Interface* **7**, 873–885 (2010). URL https://doi.org/10.1098/rsif.2009.0386.

[2] Diekmann, O., Heesterbeek, J. & Metz, J. On the definition and the computation of the basic reproduction ratio R_0 in models for infectious diseases in heterogeneous populations. *Journal of Mathematical Biology* **28** (1990). URL https://doi.org/10.1007/BF00178324.

[3] Keeling, M. J. & Rohani, P. *Modeling infectious diseases in humans and animals* (Princeton University Press, 2011). URL https://doi.org/10.2307/j.ctvcm4gk0.

[4] Funk, S. sbfnk/socialmixr (2020). Software. URL https://github.com/sbfnk/socialmixr.

[5] Mossong, J. *et al.* Social Contacts and Mixing Patterns Relevant to the Spread of Infectious Diseases. *PLoS Medicine* **5**, e74 (2008). URL https://doi.org/10.1371/journal.pmed.0050074.

	←(u)nvaccinated	←(v)accinated
u←	50.0%	13.2%
v←	26.3%	10.5%
total	76.3%	23.7%

Supplementary Table 1. Contribution to $\mathcal{R}$ from infections between vaccinated and unvaccinated groups for the "medium efficacy" scenario, considering that children and adolescents are both as susceptible and as infectious as adults.

	←(u)nvaccinated	←(v)accinated
u←	64.3%	9.8%
v←	20.6%	5.3%
total	84.9%	15.1%

Supplementary Table 2. Same as Supplementary Table 1 for lower vaccine efficacy.

	←(u)nvaccinated	←(v)accinated
u←	36.8%	17.4%
v←	28.6%	17.2%
total	65.4%	34.6%

Supplementary Table 3. Contribution to $\mathcal{R}$ from infections between vaccinated and unvaccinated groups for the medium efficacy scenario, additionally assuming that the elderly have lower protection than initially assumed.

	←(u)nvaccinated	←(v)accinated
u←	0.458	0.208
v←	0.342	0.192
total	0.8	0.4

Supplementary Table 4. Absolute contributions to $\mathcal{R}$ from infections between vaccinated and unvaccinated groups for the "medium efficacy" scenario.

	←(u)nvaccinated	←(v)accinated
u←	0.158	0.145
v←	0.215	0.345
total	0.373	0.49

Supplementary Table 5. Absolute contributions to $\mathcal{R}$ from infections between vaccinated and unvaccinated groups for the "medium efficacy" scenario, considering that vaccine uptake is 90% for adolescents, adults, and the elderly, amounting to a total vaccine uptake of 80%.

	←(u)nvaccinated	←(v)accinated
u←	27.8%	21.4%
v←	26.1%	24.7%
total	53.9%	46.1%

Supplementary Table 6. Relative contributions to $\mathcal{R}$ from infections between vaccinated and unvaccinated groups for the "very low efficacy" scenario, additionally assuming that the average effective transmission reduction for adults and elderly is $r' = 20\%$.
